# Rho-Associated Kinase Inhibitor Fasudil Protects from Sepsis-Induced Acute Kidney Injury in Rat via Suppressing STAT-3 and NLRP-3 Pathway

**DOI:** 10.3390/cimb47050340

**Published:** 2025-05-08

**Authors:** Neslihan Şahin, Ejder Saylav Bora, Osman Sezer Çınaroğlu, Oytun Erbaş

**Affiliations:** 1 Department of Emergency Medicine, Bergama State Hospital, 35700 Izmir, Turkey; 2 Department of Emergency Medicine, Faculty of Medicine, Izmir Katip Çelebi University, 35620 Izmir, Turkey; 3 Faculty of Medicine, Biruni Research Center (BAMER), Biruni University, 34015 Istanbul, Turkey; oytunerbas2012@gmail.com

**Keywords:** sepsis-associated acute kidney injury, Fasudil, STAT-3 pathway, NLRP-3 inflammasome, Rho kinase inhibitor

## Abstract

Sepsis-associated acute kidney injury (S-AKI) is a severe complication in critically ill patients, marked by inflammation, oxidative stress, and renal dysfunction. This study aimed to evaluate the renoprotective effects of Fasudil (Fas), a Rho-associated kinase inhibitor, in a rat model of S-AKI induced by cecal ligation and puncture (CLP). Thirty-six Wistar albino rats were divided into control, CLP with saline, and Fas (100 mg/kg/day intraperitoneally) groups. Biochemical, histopathological, and molecular analyses were conducted to assess kidney function, oxidative stress, and inflammation. Fas treatment significantly decreased plasma malondialdehyde and TNF-α levels, reducing oxidative stress and systemic inflammation. Kidney function markers, including BUN and creatinine, showed marked improvement. Furthermore, Fas suppressed the expression of STAT-3 and NLRP-3 in renal tissues, highlighting its role in modulating key inflammatory pathways. Histological evaluation revealed alleviated renal damage, with less tubular necrosis and interstitial inflammation in the Fas-treated group. In conclusion, Fas demonstrates significant anti-inflammatory, antioxidant, and nephroprotective effects in S-AKI, primarily by inhibiting STAT-3 and NLRP-3 signaling. These results support its potential as a therapeutic agent in sepsis-induced kidney injury and suggest the need for further clinical evaluation.

## 1. Introduction

Sepsis-associated acute kidney damage (S-AKI) is a common and sometimes lethal consequence in hospitalized critically unwell patients. Recent progress in understanding the underlying mechanisms and the development of biomarkers has enhanced the accuracy of diagnosis [1]. Additionally, it is the prevailing AKI syndrome observed in intensive care units (ICUs), where prompt resuscitation and careful administration of fluids and vasoactive drugs are essential for both prevention and treatment [2]. S-AKI is a complex mitochondrial reaction that causes inflammation, oxidative damage, microvascular dysfunction, and tubular cell maladaptation to septic injury [3].

AKI refers to the temporary decline in kidney function, which includes mild acute renal failure [4]. Two main phenomena highlight the seriousness of AKI. Initially, it is important to note that AKI has significant ramifications, and if AKI occurs repeatedly, it can lead to the development of chronic kidney disease [5]. Furthermore, the prevalence of AKI is significantly high and has been observed to be increasing over time [6,7]. Efforts to prevent, diagnose, and treat AKI at an early stage have received significant attention.

The Rho kinase pathway is essential for the architecture and functionality of several kidney cell types, including tubular epithelial cells, mesangial cells, and podocytes [8]. The Rho/Rho kinase pathway controls the constriction of renal arterioles, which in turn affects renal blood flow and filtration rate [8]. Recently, researchers have created potent and targeted inhibitors of Rho kinase, which have been tested for their curative properties on various models of renal injury [9]. Treatment with novel Rho kinase inhibitors reduces the development of renal damage in experimental studies [10,11,12].

Fasudil (Fas) is a widely used blocker of the Rho enzymes that effectively blocks the activity of several protein kinases. Currently, it is employed as a cutting-edge cerebrovascular vasodilator [10]. Research has demonstrated that the Rho enzyme can potentially induce harm to the renal tubules [9]. Fas, a specific inhibitor of the Rho enzyme, has demonstrated its ability to protect against renal fibrosis [11,12]. Additionally, Fas can prevent the transformation of renal tubular epithelial cells into myofibroblasts, which is caused by excessive sugar-induced differentiation of renal tubular epithelial fibroblasts [13,14]. Fas can also aid in decelerating the progression of chronic renal failure, although its distinct mechanisms have been increasingly elucidated [15].

In light of these issues, this study aims to investigate the protective effects of the Rho-associated kinase inhibitor Fas on sepsis-induced Acute Kidney Injury (AKI) in a rat model. We aim to determine whether Fas suppresses the STAT-3 and NLRP-3 pathways, which are crucial to sepsis-related inflammation and immune responses.

## 2. Materials and Methods

### 2.1. Animal Care

Thirty-six adult female Wistar albino rats with a weight range of 200 to 250 g were employed in this study. The Animal Ethics Committee of Science University (Ethical number 2924060301) approved all procedures, which complied with the National Institutes of Health (USA) guidelines for the care and use of laboratory animals. The rats were obtained from the Experimental Animal Laboratory at Science University. The animals were maintained in steel enclosures featuring a 12 h light/dark cycle, controlled ambient conditions (22 ± 2 °C), and unrestricted access to food. The study was conducted by the Basic and Clinical Pharmacology and Toxicology policy for experimental and clinical studies [16].

### 2.2. Method

We randomly assigned 36 rats into two groups to establish a sepsis model (intervention groups) and utilized the cecal ligation and puncture (CLP) technique. The study groups were structured in the following manner:

Twelve rats were used as samples and received food via oral administration.

Twelve rats underwent surgical procedures, receiving intraperitoneal administration of 0.9% sodium chloride.

Twelve rats in Group 3 received surgical intervention. After this, an intraperitoneal injection of 100 mg/kg/day of Fas was administered.

The procedures for groups 2 and 3 were the same. During the first hour after surgery, 15 mL/kg of 0.9% NaCl was administered intraperitoneally every 12 h, followed by daily 10 mL/kg/day of 0.9% NaCl.

The investigation spanned five days. Seven rats died. The cohort administered CLP and water recorded five fatalities, whereas the cohort receiving CLP and Fas noted two fatalities. Blood samples were collected via heart puncture for chemical analysis following the euthanasia of the animals at the end of the experiment. The kidneys were also extracted for histopathological examination.

A 3 cm incision was made in the midsection of the abdomen to access the cecum during the CLP procedure. The cecum was sutured to the iliocecal valve with a 3.0 silk suture, and a 22-gauge needle was utilized to establish a single opening in the cecum. This intervention facilitated the expulsion of a limited amount of excrement. The cecum was reinserted into the abdomen, and the incision was closed using 4-0 polyglactin sutures. The rats in the placebo group underwent laparotomy with no binding or puncturing of their cecum. Sepsis onset was observed five hours post-CLP completion [17].

### 2.3. Renal Histopathological Analysis

The kidneys were excised and maintained in 10% formaldehyde in 0.1 M PBS for three days. The Olympus C-5050 digital camera (Olympus Corp., Tokyo, Japan) was used to photograph H&E-stained slices using an Olympus BX51 microscope (Olympus Corp., Tokyo, Japan). Unaware of group designations, the evaluator examined 10 microscopic fields per slice at ×20 magnification using a computerized image analysis system (Image-Pro Express 1.4.5, Media Cybernetics, Inc., Rockville, MD, USA).

A semi-quantitative scale test was assessed by evaluating tubular epithelial necrosis, luminal necrotic debris, tubular dilation, bleeding, and interstitial inflammation in kidney sections from each rat. The scale has five categories: 0 (0–5%), 1 (6–20%), 2 (21–40%), 3 (41–60%), 4 (61–80%), and 5 (81–100%).

### 2.4. Hematological Analysis

Blood samples were obtained by heart puncture and placed in heparinized containers with a 1 mL syringe. The plasma was kept at −20 °C until analysis after centrifugation at 3000 rpm for 10 min at room temperature. A Beckman-Coulter AU 640 auto-analyzer was used to determine blood urea nitrogen (BUN) and creatinine levels, along with commercial samples acquired from Beckman-Coulter Inc. in Brea, CA, USA.

### 2.5. Renal Biochemical Analysis

The organs were stored at −20 °C after decapitation before biochemical examination. Before centrifugation at 5000× *g* for fifteen minutes, the kidney tissue was homogenized five times with PBS (pH 7.4) using a glass homogenizer. Bradford’s method measured the protein concentration in the supernatant, using bovine serum albumin as the standard. The concentrations of STAT-3 and NLRP-3 in the kidney tissue supernatants were quantified utilizing commercially available rat ELISA kits.

### 2.6. Quantification of TNF-α Concentrations in Plasma

The manufacturer’s guidelines quantified Plasma TNF-α concentrations with enzyme-linked immunosorbent assay (ELISA) kits from Biosciences, Abcam, Cambridge, UK.

### 2.7. Assessment of Lipid Peroxidation

Malondialdehyde (MDA) levels were quantified as thiobarbituric acid reactive substances (TBARS) to assess the lipid peroxidation that transpired in plasma. Plasma samples were combined with trichloroacetic acid and TBARS reagents, heated to 100 °C for 60 min, cooled on ice, and centrifuged at 3000 revolutions per minute for 20 min. The absorbance of the supernatant was quantified at 535 nm, and the concentrations of MDA were reported in nanomoles. Tetraethoxypropane was employed for the calibration procedure [18].

### 2.8. Statistical Analysis

Unless otherwise stated, all quantitative data were expressed as mean ± standard error of the mean (SEM). Before inferential analysis, the assumption of normality was assessed using the Shapiro–Wilk test and the homogeneity of variances was evaluated using Levene’s test. Since assumptions of parametric tests were met, one-way analysis of variance (ANOVA) was used to compare mean values across multiple groups for continuous outcomes such as biochemical and histopathological parameters. Where overall group differences were statistically significant (*p* < 0.05), Bonferroni-corrected post hoc pairwise comparisons were conducted to identify specific intergroup differences while controlling for Type I error inflation. The Kaplan–Meier survival estimator was used for categorical survival data, and statistical significance between survival distributions was tested using the Log-Rank (Mantel–Cox) test. The significance level for all statistical tests was set at *p* < 0.05 (two-tailed). Cohen’s Kappa coefficient was also calculated to assess the consistency and reliability of histopathological scoring performed by two independent blinded observers. The resulting κ value was 0.87, indicating strong inter-rater agreement and supporting the robustness of the histological evaluations. Effect sizes were also interpreted where relevant to provide additional context for clinical or biological relevance, particularly in comparisons of treatment and control groups. All statistical analyses were performed using SPSS Statistics v26.0 (IBM Corp., Armonk, NY, USA) and GraphPad Prism v9.0 (GraphPad Software Inc., San Diego, CA, USA) for statistical computing and graphical presentation.

## 3. Results

### 3.1. Mortality and Survival

**Figure 1:**
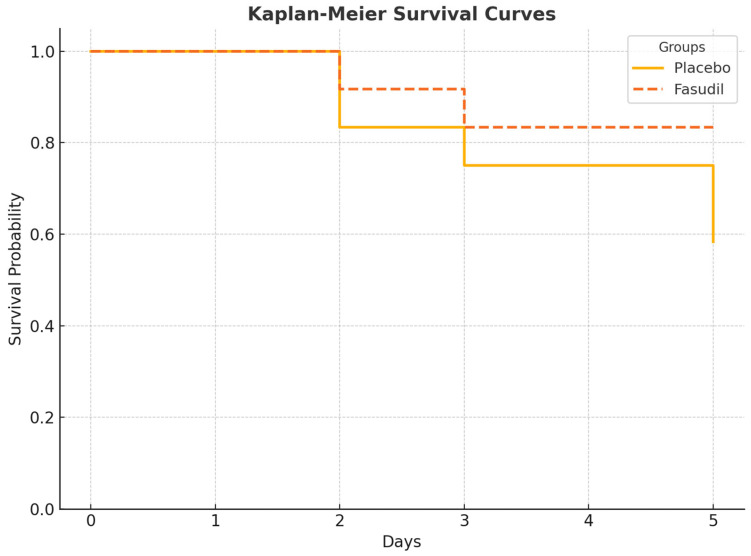
Kaplan–Meier survival curves for the three groups over 5 days. Statistical comparison is done using the Log-Rank (Mantel–Cox) test.

As shown in Figure 1, the survival rate of rats in the CLP and saline group significantly declined during the 5-day observation period (only 7/12 survived). In contrast, the Fasudil-treated group showed improved survival, with 10/12 rats surviving to the study endpoint. The survival rate in the control group was 100%. The differences between the groups were statistically significant (Log-Rank test, *p* < 0.05).

### 3.2. Biochemical Parameters

Biochemical analysis results (Table 1) demonstrated significant plasma and kidney marker alterations due to sepsis-induced acute kidney injury (AKI). Plasma malondialdehyde (MDA), an oxidative stress marker, was significantly elevated in the CLP and saline group (144.2 ± 9.5 nM) compared to the normal control group (61.8 ± 10.1 nM, ** *p* < 0.001 **). Treatment with Fas reduced MDA levels (107.6 ± 12.5 nM, # *p* < 0.01). Similarly, plasma tumor necrosis factor-alpha (TNF-α), a pro-inflammatory cytokine, was markedly increased in the CLP and saline group (185.1 ± 12.6 pg/mL, ** *p* < 0.001), while Fas treatment significantly decreased TNF-α levels (89.5 ± 9.7 pg/mL, ## *p* < 0.001).

Markers of renal function, including plasma blood urea nitrogen (BUN) and creatinine, were significantly elevated in the CLP and saline group compared to controls (48.4 ± 1.9 mg/dL vs. 24.5 ± 1.3 mg/dL and 0.57 ± 0.08 mg/dL vs. 0.29 ± 0.1 mg/dL, respectively, ** *p* < 0.05 or ** *p* < 0.001). Fas treatment attenuated these increases (BUN: 34.2 ± 2.3 mg/dL, creatinine: 0.43 ± 0.2 mg/dL, # *p* < 0.05).

In the kidney tissue, STAT-3 and NLRP-3 protein levels were significantly elevated in the CLP and saline group compared to the control (STAT-3: 2.86 ± 0.3 pg/mg vs. 0.93 ± 0.12 pg/mg, ** *p* < 0.001; NLRP-3: 125.1 ± 5.4 pg/g vs. 49.4 ± 3.7 pg/g, ** *p* < 0.001). Fas treatment significantly reduced both STAT-3 (1.75 ± 0.28 pg/mg, ## *p* < 0.001) and NLRP-3 (98.2 ± 7.7 pg/g, # *p* < 0.01) (Table 1).

### 3.3. Histopathological Analysis

**Figure 2:**
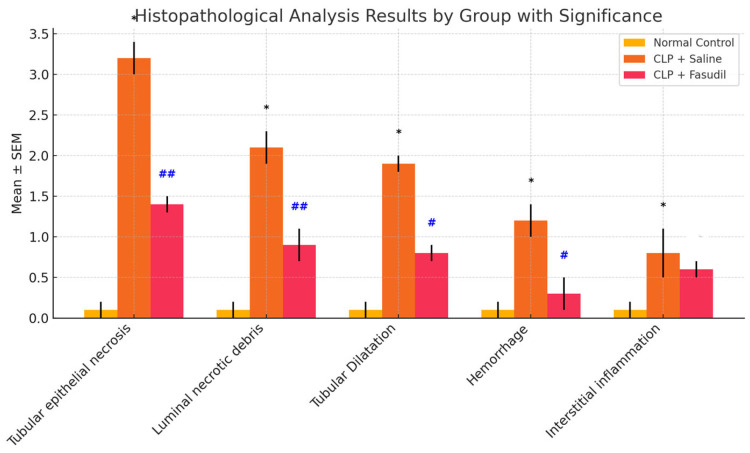
Histopathological evaluations of the kidney are graphically presented. Results were presented as mean ± SEM. Statistical analyses were performed using one-way ANOVA and a post hoc Bonferroni test. * *p* < 0.001, different from normal groups; # *p* < 0.05, ## *p* < 0.001 different from CLP and saline group.

Histopathological evaluation (Figure 2) revealed significant tubular epithelial necrosis, luminal necrotic debris, tubular dilatation, hemorrhage, and interstitial inflammation in the CLP and saline group compared to the normal control group (** *p* < 0.001). Fas treatment ameliorated these changes significantly, with marked reductions in tubular epithelial necrosis (1.4 ± 0.1 vs. 3.2 ± 0.2, ## *p* < 0.001), luminal necrotic debris (0.9 ± 0.2 vs. 2.1 ± 0.2, ## *p* < 0.001), and tubular dilatation (0.8 ± 0.1 vs. 1.9 ± 0.1, # *p* < 0.05).

**Figure 3:**
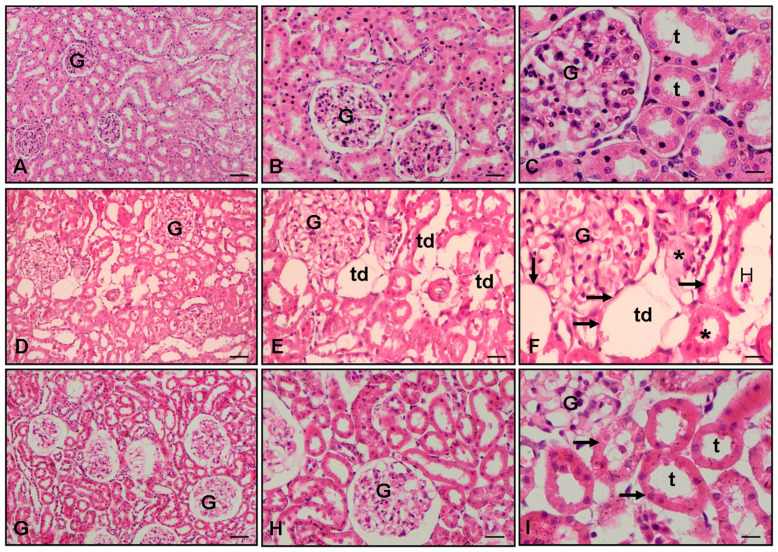
Kidney histopathology ×10, ×20, and ×40 magnification, hematoxylin and eosin (H&E) stain. (**A**–**C**) Normal group kidney, renal tubules (t); glomerulus (G); (**D**–**F**) CLP and saline groups showed severe histopathologic alteration showed kidney injury for tubular dilatation (td), tubular epithelial necrosis (arrow), hemorrhage (H), and luminal necrotic debris (asterisks); (**G**–**I**) CLP and 100 mg/kg Fasudil groups showed decreased injury for normal tubules (t) and tubular epithelial cells (arrow).

Representative histological pictures of the kidney (Figure 3) further demonstrate the protective effects of Fas. The CLP and saline group had pronounced histopathologic changes, including tube damage and necrosis, which were markedly reduced in the Fas-treated group.

In the normal control group, histopathological scores for all parameters (tubular epithelial necrosis, luminal necrotic debris, tubular dilatation, hemorrhage, and interstitial inflammation) were consistently reported as 0.1 ± 0.1. This uniformity resulted from 10 out of 12 rats scoring 0 (no histopathological abnormalities) and two scoring 1 (minimal focal changes), reflecting normal biological variability in healthy animals. All evaluations were performed in a blinded manner by two independent observers. The inter-rater agreement was high (Cohen’s kappa κ = 0.87), supporting the reliability of the scoring process.

## 4. Discussion

Sepsis-associated acute kidney injury (S-AKI) is characterized by a rapid decline in renal function, damage to tubular epithelial cells, and the accumulation of inflammatory cytokines in the kidney, often culminating in multiorgan failure. Understanding the pathophysiology of S-AKI is essential for developing effective treatments [1]. Although acute kidney injury (AKI) is a complex and prevalent condition, various therapeutic approaches have shown increasing efficacy in the last decade [19]. This study evaluated the protective and reparative effects of Fas—particularly its anti-inflammatory, antioxidant, and anti-apoptotic properties—on AKI.

Previous studies have shown that Fas exhibits beneficial effects in nephrotoxicity models induced by contrast agents or doxorubicin, including anti-inflammatory, anti-apoptotic, and antioxidant actions. Additionally, it modulates lipid and amino acid metabolism to reduce kidney injury [20,21,22]. However, unlike previous models, our study used a sepsis-induced AKI model. The anti-inflammatory effect of Fas is believed to involve the modulation of macrophage polarization—from pro-inflammatory M1 to anti-inflammatory M2 phenotypes—as seen in diabetic nephropathy models [23]. Given the critical role of macrophages in renal inflammation, this model may also apply to S-AKI.

Moreover, advanced multiparametric MRI studies have begun quantifying the renoprotective effects of Fas in vivo, offering noninvasive tools to monitor its therapeutic impact [21]. These innovations help bridge the gap between experimental research and clinical practice.

A study by Hong et al. demonstrated that high-dose Fas protected cirrhotic rats from hepatic ischemia/reperfusion injury by downregulating HIF-1α, upregulating superoxide dismutase (SOD), and reducing MDA and ET-1 levels—even at a dose as low as 10 mg/kg [24]. Similarly, our study observed reduced MDA levels following Fas administration (100 mg/kg/day). Li et al. reported that Fas inhibits the production of TNF-α, IL-1, and NO in LPS-stimulated microglia, possibly by suppressing the TLR4 pathway [25]. In our study, TNF-α levels were significantly lower in the Fas-treated group. Improvements in BUN and creatinine levels also indicate a renal protective effect.

Zhang et al. demonstrated Fas’s effectiveness in contrast-induced renal insufficiency by regulating renal hemodynamics and relaxing vascular smooth muscle [26]. Wang et al. attributed these protective effects to Fas’s inhibition of the Rho/ROCK pathway, which blocks inflammation, apoptosis, and oxidative damage in renal tissues [21]. Our findings align with these mechanisms, primarily through the observed suppression of key immune regulatory proteins such as STAT-3 and NLRP3. Inhibition of STAT-3 is particularly important, as it plays a central role in cytokine signaling during sepsis. Likewise, inhibition of NLRP3 inflammasome activation suggests Fas may reduce pyroptosis—an inflammatory form of cell death that worsens organ dysfunction in sepsis [27].

Similarly, Hassanein et al. showed that umbelliferone reduces gentamicin-induced renal injury by inhibiting the NLRP3 and STAT-3 pathways [28]. Our study shows that Fas also suppressed both pathways and cleaved caspase-3 expression.

Fas’s renoprotective effects may also stem from its ability to regulate the Akt/eNOS/NO pathway, reduce apoptosis, and enhance autophagy [29]. Its suppression of the NLRP3 inflammasome and promotion of mitophagy further underscore its role in minimizing inflammation and cellular damage [30].

In line with previous Fas studies [22,23,24], our histopathological findings show that kidney tissue damage—especially at the tubular level—was alleviated after treatment. Reduced tubular necrosis and interstitial inflammation suggest that Fas effectively preserves renal architecture. Though we did not directly investigate glomerular changes or podocyte health, findings from Tian et al. demonstrate Fas’s ability to stabilize actin cytoskeleton dynamics and regulate YAP signaling in podocytes by inhibiting Rho GTPase activity [31]. These upstream effects may contribute to broader renoprotective benefits, including tubular and glomerular compartments.

In a separate study, Suxia et al. found that Fas alleviates sepsis-induced AKI in rats by reducing endothelin-1 expression and inhibiting Rho kinase signaling [32]. Similarly, Demeng et al. observed that Fas mitigates early kidney damage in a cisplatin-induced injury model via lipid and amino acid metabolism regulation [33].

In conclusion, our data strongly support Fas’s therapeutic potential for S-AKI. Its multimodal effects—including suppression of the Rho/ROCK pathway, inhibition of oxidative and inflammatory cascades, stabilization of cellular structures, and immune modulation—make it a promising agent for future clinical applications. Targeting intracellular regulators like STAT-3 and NLRP3 may be crucial for controlling immune dysregulation and preserving renal function in septic conditions.

### Limitations

As this is an experimental study for use in humans, more studies are needed. Moreover, 12 h, 24 h, and 48 h will provide a better understanding of injury dynamics and therapeutic response. We were not able to use biomarkers such as NGAL/KIM-1, IL-1β, and IL-6, and pro-apoptotic markers like PARP-1 and caspase 3, 8, and 9 despite knowing their importance due to budget constraints and the availability of analysis during the study.

## 5. Conclusions

This study demonstrates that Fas effectively mitigates inflammation, decreases oxidative stress, and maintains renal function in a rat model of S-AKI by inhibiting the STAT-3 and NLRP-3 signaling pathways. The findings indicate that Fas may serve as a new therapeutic option for the management of sepsis-induced renal injury. Additional research is necessary to investigate its applicability in clinical environments, particularly when integrated with advanced monitoring methods to enhance therapeutic effectiveness in real time.

## Figures and Tables

**Table 1:** Biochemical analysis results were presented as mean ± SEM. Statistical analyses were performed using one-way ANOVA. * *p* < 0.05, ** *p* < 0.001 different from normal groups; # *p* < 0.01, ## *p* < 0.001 different from CLP and saline group.

	Normal Control	CLP and Saline Group	CLP and 100 mg/kg Fasudil (Fas) Group
Plasma MDA (nM)	61.8 ± 10.1	144.2 ± 9.5 **	107.6 ± 12.5 #
Plasma TNF alfa (pg/mL)	12.8 ± 3.3	185.1 ± 12.6 **	89.5 ± 9.7 ##
Plasma BUN (mg/dL)	24.5 ± 1.3	48.4 ± 1.9 **	34.2 ± 2.3 #
Plasma Creatinin (mg/dL)	0.29 ± 0.1	0.57 ± 0.08 *	0.43 ± 0.2 #
Kidney STAT-3 (pg/mg protein)	0.93 ± 0.12	2.86 ± 0.3 **	1.75 ± 0.28 ##
Kidney NLRP-3 (pg/g protein)	49.4 ± 3.7	125.1 ± 5.4 **	98.2 ± 7.7 #

## Data Availability

Data are available upon reasonable request from the corresponding author.
